# A case of mirror syndrome caused by hydrops fetalis after fetoscopic laser photocoagulation

**DOI:** 10.1002/ccr3.1513

**Published:** 2018-04-06

**Authors:** Emi Kino, Yohei Maki, Masanao Ohhashi, Seishi Furukawa, Takatsugu Maeda, Hiroshi Sameshima

**Affiliations:** ^1^ Department of Obstetrics and Gynecology Faculty of Medicine University of Miyazaki Miyazaki Japan; ^2^ Department of Obstetrics and Gynecology Kagoshima City Hospital Kagoshima Japan

**Keywords:** Fetoscopy, hydrops fetalis, mirror syndrome, pregnancy, twin‐to‐twin transfusion syndrome

## Abstract

Fetoscopic laser photocoagulation (FLP) of placental anastomoses is a well‐established procedure for twin‐to‐twin transfusion syndrome that improves fetal outcome with rare maternal complications. However, fetal hydrops can develop even after FLP, and mirror syndrome can occur, indicating that both the fetal and maternal courses should be monitored after FLP.

## Introduction

A woman with a monochorionic diamniotic twin pregnancy developed twin‐to‐twin transfusion syndrome and underwent fetoscopic laser photocoagulation (FLP). The ex‐donor fetus developed hydrops fetalis on day 3 after FLP and the woman developed mirror syndrome on day 21. This case shows fetal hydrops leading mirror syndrome can occur after FLP.

Fetoscopic laser photocoagulation (FLP) of placental anastomoses is a well‐established procedure for twin‐to‐twin transfusion syndrome (TTTS) that improves fetal mortality and neurodevelopmental outcome 1. Major procedure‐related complications include preterm premature rupture of membranes, preterm labor, placental abruption, vascular laceration, and membrane laceration 1. Severe maternal complications are rare, but occur in 1.8% of cases 2.

Mirror syndrome is characterized by maternal edema associated with hydrops fetalis and placental edema. Its etiology is poorly understood, although both immune and nonimmune hydrops can cause mirror syndrome. TTTS, a serious complication of monochorionic diamniotic (MD) twinning, occurs in approximately 10% of MD twins 1; it is an important cause of hydrops fetalis responsible for 18% of cases of mirror syndrome 3. We report here a case of mirror syndrome caused by hydrops fetalis occurring after FLP.

## Case Report

A 31‐year‐old gravida 1, para 1 woman was diagnosed with a MD twin pregnancy at 15 gestational weeks. She had no complications before pregnancy and no family history of genetic diseases. Her previous pregnancy was uneventful. At 22 weeks, severe oligohydramnios (Maximum vertical pocket [MVP] of 1.4 cm) and absence of bladder filling were found in fetus A, and polyhydramnios (MVP of 10 cm) was found in fetus B. Fetal Doppler blood flow velocity waveforms analysis in the umbilical arteries revealed intermittently absent flow in fetus A. TTTS stage II was diagnosed and the woman was referred to Kagoshima City Hospital for FLP. FLP was conducted at 22 57 weeks under spinal anesthesia, and all visible anastomoses were coagulated. Follow‐up ultrasound examinations on day 3 after FLP revealed that fetus A (ex‐donor) had developed hydrops fetalis with skin edema, pleural effusion, and ascites; MVP of fetus A was 2–3 cm. MVP of fetus B (ex‐recipient) was 7–8 cm with no sign of hydrops fetalis. Fetal Doppler findings on both fetuses were normal. Hydrops fetalis of fetus A gradually worsened. The patient was aware of decreased urine output at 25 2/7 weeks (18 days after FLP).

**Figure 1:**
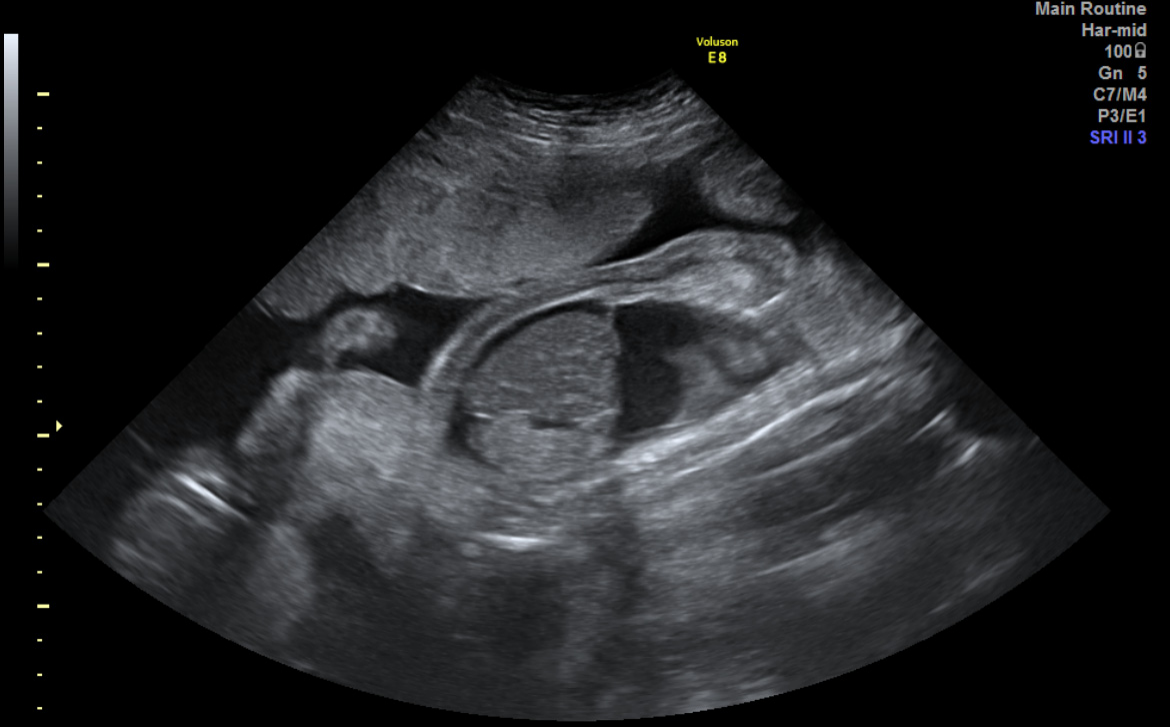
Ultrasound examinations on day 21 after FLP showed severe hydrops fetalis with skin edema, pericardial effusion, and ascites in fetus A (ex‐donor). Edematous placenta was also noted.

She returned to our hospital at 25 5/7 weeks (21 days after FLP). Ultrasound examinations found oligohydramnios (MVP < 2 cm) and severe hydrops fetalis with skin edema, pleural effusion, pericardial effusion, and ascites in fetus A (ex‐donor) (Fig. 1). The placenta was also edematous. Fetus B (ex‐recipient) had a MVP of 8.7 cm and pleural effusion. Fetal Doppler blood flow velocity waveforms analysis in the umbilical arteries revealed absent flow in fetus A, but normal in fetus B. The cardio‐thoracic area ratio (CTAR) in fetus A was only 15% with poor wall motion. The mother was afebrile with a blood pressure of 125/69 mmHg, pulse rate of 92 bpm, and SpO_2_ of 98% on room air. She had systemic edema with weight gain of 4.5 kg in 2 weeks. Laboratory studies showed the following data: anemia (hemoglobin, 9.7 g/dL; hematocrit, 29.4%); hypoalbuminemia (albumin, 2.17 mg/dL); platelet count, 220,000/mm^3^; white cell count, 6300/mm^3^; Na, 138 mEq/L; K, 5.3 mEq/L; uric acid, 7.9 IU/L; urea nitrogen, 19.9 mg/dL, elevated levels of serum creatinine (1.0 mg/dL); 24‐h urine protein, 339 mg; slightly elevated levels of liver enzymes (AST, 53 IU/L; ALT, 27 IU/L; LDH, 393 IU/L). Coagulation studies, including antithrombin III of 77%, were normal. Serum hCG level was elevated at 167,199 mIU/mL. On the evening of admission, she complained of dyspnea and her SpO_2_ dropped to 89%. Arterial blood gas values were as follows: pH, 7.49; PaO_2_, 58.1 mmHg; PaCO_2_, 29.4 mmHg; HCO_3_, 22.1 mmol/L; base excess, −0.9. Chest radiography showed bilateral ground‐glass appearance, indicating pulmonary edema. We diagnosed mirror syndrome; therefore, we performed a cesarean section under general anesthesia. There were no complications associated with the cesarean section. She was admitted to the intensive care unit and treated with carperitide and furosemide under synchronized intermittent mandatory ventilation. Ultrasound cardiography showed normal cardiac function. After 2 days of oliguria and a peak serum creatinine level of 1.8 mg/dL, she was extubated and discharged from the hospital 11 days after delivery.

Female infant A (ex‐donor) was live born weighing 1524 g with an Apgar score of 1 at 1 min and 3 at 5 min. Umbilical artery blood gas analysis showed a pH of 7.32, and low albumin level (0.7 mg/dL), and elevated BNP level (>2000 pg/mL), but no anemia (hematocrit, 45%) at birth. She did not respond to intensive care and died 20 h after birth. Autopsy findings showed a hypoplastic heart compared with that of normal fetuses in the same gestational age with severe liver congestion indicating congestive heart failure. Female infant B (ex‐recipient) was live born weighing 758 g with an Apgar score of 2 at 1 min and 5 at 5 min. Umbilical artery blood gas analysis showed a pH of 7.41. Hematocrit was 42%. She was incubated and received surfactant administration. Polyuria and hypotension were treated with catecholamines and volume loading. Her neonatal course was uneventful, and she was discharged on day 180. She exhibited psychomotor retardation at 2 years old.

The examination of dyed milk injection into umbilical vessels of each twin showed there was no remaining anastomosis.

## Discussion

FLP has been established as a first line therapy for TTTS between 16 and 26 weeks of gestation, with improved perinatal mortality from 70–100% to 30–50% and a 5–20% risk of long‐term neurodevelopment disability 1. Data on maternal complications of FLP are insufficient, but complications are considered rare. The prevalence of severe maternal complications, including pulmonary and surgical sequelae, placental abruption, and ICU admission, was reported to be 1.8% 2. Therefore, FLP is thought to be an effective procedure for TTTS with good maternal safety. However, the present case shows that fetal hydrops can develop even after FLP, and mirror syndrome can subsequently occur, indicating that both the fetal and maternal courses should be carefully monitored after FLP.

As maternal symptoms associated with mirror syndrome disappear shortly after the successful treatment of hydrops fetalis or delivery, whether hydrops fetalis improves is important in determining the timing of delivery. There are six cases, including the present case, that report mirror syndrome occurring after FLP (Table 1) 4, 5, 6, 7, 8. In cases 1, 2, and 3, preexisting hydrops fetalis promptly improved after FLP, maternal symptoms also improved, and pregnancy was successfully prolonged. On the other hand, in cases 4, 5, and our case, hydrops fetalis developed after FLP following severe maternal complications that resulted in preterm delivery.

**Table 1:** Six cases of mirror syndrome occurring after fetoscopic laser photocoagulation

Case	GA at diagnosis of TTTS	Quintero Stage	GA at FLP	Hydrops fetalis		Time of diagnosis of mirror syndrome	Maternal course of mirror syndrome	GA at delivery	Neonatal outcome		Ref
				Donor	Recipient				Donor	Recipient	
1	21	IV	21	None	+, improved after FLP	Post‐FLP day 4	Improved after FLP day 10	37	Alive	Alive	4
2	23	III	24	None	+, improved after FLP	Post‐FLP day 12	Improved after FLP day 12	36	Alive	Alive	5
3	23	IV	23	None	+, improved after FLP	Post‐FLP day 1	Improved after delivery day 10	34	Alive	Alive	6
4	24	IV	25	+, developed after FLP	IUFD	On the day of FLP	Improved after delivery day 2	25	Alive	IUFD	7
5	26	IV	27	+, developed after FLP	+, improved after FLP	N/A (27 weeks)	Improved after delivery	27	Neonatal death	Alive	8
Present case	22	III	22	+, developed after FLP	None	Post‐FLP day 21	Improved after delivery day 2	25	Neonatal death	Alive	

GA, gestational age; TTTS, twin‐to‐twin transfusion syndrome; FLP, fetoscopic laser photocoagulation; IUFD, intrauterine fetal death.

One study showed that 27% (10/40) of donors developed one or more hydropic signs, and 90% (9/10) of them were mild or moderate and transient, while one donor fetus died of severe hydrops 9. The presumed hemodynamic changes are likely to play a major role in the development of hydrops fetalis in the donor fetus after FLP. In the present case, the clinical and autopsy findings of the ex‐donor infant showed a hypoplastic heart and congestive heart failure. We support the hypothesis that the ex‐donor infant with a hypoplastic heart was unable to tolerate prompt volume loading in utero due to the shutdown of anastomoses by FLP. We did not check the cardiac condition in the donor fetus before FLP, as a donor fetus rarely has cardiac failure, not like a recipient fetus, in a case of TTTS. Our case indicates that serial ultrasonographic examination for a cardiac function in a donor fetus should be carried out before and after FLP, although whether hypoplastic heart leading hydrops fetalis after FLP can be predicted is unknown. We also presume that hypoalbuminemia exacerbated the condition.

The pathophysiology of mirror syndrome remains unknown. Some reports suggest that high levels of hCG produced by placental edema play an important role in the development of mirror syndrome 4, 7, 10. The elevated hCG level in our case supports this hypothesis. Serum hCG level was reported to be a useful maker for evaluating the efficacy of FLP for TTTS, as it decreases with successful treatment. In addition, a case report of mirror syndrome after FLP showed that hCG level increased after the procedure. Unfortunately, we did not evaluate hCG level before and after FLP, but hCG might be a predictive marker for mirror syndrome.

In summary, we report a case of mirror syndrome associated with hydrops fetalis developing after FLP. Careful management is required even after FLP, especially in cases of hydrops fetalis that develops after the procedure.

## Authorship

EK: drafted the manuscript. YM: edited the manuscript. MO: collected clinical data. SF and TM: provided additional comments. HS: supervised the whole process. All authors read and approved the final version of manuscript.

## Conflict of Interest

No author has any potential conflict of interest relevant to this manuscript.
